# Remote Psychotherapy During the COVID-19 Pandemic. Experiences With the Transition and the Therapeutic Relationship. A Longitudinal Mixed-Methods Study

**DOI:** 10.3389/fpsyg.2021.743430

**Published:** 2021-11-24

**Authors:** Robert Stefan, Gerd Mantl, Claudia Höfner, Julia Stammer, Markus Hochgerner, Kathrin Petersdorfer

**Affiliations:** Österreichischer Arbeitskreis für Gruppentherapie und Gruppendynamik, Vienna, Austria

**Keywords:** remote psychotherapy, COVID–19, psychotherapeutic relationship, mixed method approach, videotherapy

## Abstract

**Aims:** Research conducted prior to the onset of the COVID-19 pandemic indicates that remote psychotherapy is as effective as in-person treatment. At that time, it usually was the therapist’s individual choice to work remotely, whereas the pandemic pushed psychotherapists, including previous skeptics, to incorporate remote work methods into their routine due to limited face-to-face contact. There is little knowledge of the way therapists experienced this sudden and forced transition to remote psychotherapy as the only treatment option. The present study aims to assess psychotherapists’ experience and proficiency delivering remote psychotherapy as well as to investigate perceived changes in the psychotherapeutic relationship.

**Methods:** An online survey was administered to psychotherapists of the Austrian Association for Group Therapy and Group Dynamics (ÖAGG). Three test periods (t) were set (t1: April, 2020 with *N* = 175; t2: May–June, 2020 with *N* = 177; t3: November–December, 2020 with *N* = 113). Research was conducted longitudinally using a mixed-methods research design.

**Results:** While psychotherapists’ levels of experience with telephone-based psychotherapy remained similar across all test periods, they became slightly more experienced using video therapy over the test period observed. However, they continued to feel less experienced compared to the use of telephone-based psychotherapy. The therapeutic relationship appeared to improve over the course of the first two test periods, while the third period showed a slight decline. No general deterioration of the psychotherapeutic relationship was found in the timespan studied.

**Conclusion:** Despite many challenges and concerns, psychotherapists seem to adapt and enhance their skills in remote psychotherapy over time. The present paper confirms and enhances previous findings in the field due to its longitudinal approach. Remote psychotherapy can be a credible and trustworthy alternative to in-person treatment to be adopted and implemented on principle by a majority of psychotherapists regardless of their orientation. Furthermore, it sheds light on chances, problems und general observations regarding the comprehensive provision of remote psychotherapy in a pandemic situation.

## Introduction

The onset of the COVID-19 pandemic sparked an unprecedented embrace of virtual health care technologies (Webster, 2020; Wind et al., 2020). Similar to most countries around the world, the first COVID-19 lockdown in Austria was imposed in mid-March 2020. The public healthcare system, which had not previously covered remote psychotherapy, quickly and unbureaucratically introduced partial reimbursement of remote psychotherapy (ÖBVP, 2020). Previously viewed with skepticism by many psychotherapists (Connolly et al., 2020), remote psychotherapy suddenly became routine practice for ongoing and new psychotherapies (Probst et al., 2020; Höfner et al., 2021a; for an overview see Wind et al., 2020). Research conducted prior to the onset of the COVID-19 pandemic indicates that remote psychotherapy is as effective as in-person treatment (Barak and Grohol, 2011). The efficacy of cognitive-behavioral approaches in remote psychotherapy is supported by many trials and respective meta-analyses (Mohr et al., 2008, 2012; Carlbring et al., 2018; Poletti et al., 2020). According to reviews by Poletti et al. (2020) and Markowitz et al. (2021), fewer data are available on psychodynamic, humanistic-existential or systemic psychotherapeutic approaches. In Austria, all of the above are equally accredited with the health care system (BMSGPK, 2020). The pandemic presented a unique opportunity to investigate how psychotherapists of various orientations dealt with the forced shift to remote psychotherapy, as numerous publications already show (Humer et al., 2020; Korecka et al., 2020; Höfner et al., 2021b; Mantl et al., 2021; Probst et al., 2021). The present study examined psychotherapists’ experience of the sudden transition to remote psychotherapy and how possible changes to the psychotherapeutic relationship were perceived between March and November 2020. Psychotherapists of humanistic, psychodynamic and systemic orientations participated in this study.

## Aims

The first aim was to quantitively assess psychotherapists’ levels of experience with the transition to remote psychotherapy and how capable they felt in its delivery during the pandemic. The study secondly aimed to qualitatively investigate perceived changes to the psychotherapeutic relationship by posing the open question “What changes in the therapeutic relationship do you perceive with the use of remote psychotherapy?” Research was conducted longitudinally with three test periods using a mixed-methods research design in order to cover both areas of interest.

## State of the Art

### Remote Psychotherapy

Despite considerable skepticism by many clinicians and patients (Connolly et al., 2020), mounting empirical evidence over the last three decades points to the effectiveness of remote psychotherapy and the emerging body of studies is very promising for most clinical conditions (Mohr et al., 2012; Carlbring et al., 2018; Swartz, 2020). Most research considers remote psychotherapy to be roughly equivalent to in-person treatment in its efficacy (Sucala et al., 2012; Poletti et al., 2020). As a limitation, it is frequently mentioned that participants in surveys on remote psychotherapy might be more computer-savvy and have a positive attitude toward remote psychotherapy; accordingly, this may lead to a positive bias as far as results are concerned (Markowitz et al., 2021).

As with most psychotherapy research, literature on remote psychotherapy is dominated by the cognitive-behavioral field (Poletti et al., 2020; Markowitz et al., 2021). Weinberg (2020) assumes that cognitive-behavioral forms of treatment are better suited for remote psychotherapy than treatments which focus on interaction and the psychotherapeutic relationship. According to Ogden and Goldstein (2020), relational therapist-patient interaction, especially non-verbal processes, which can largely be missing in remote psychotherapy, play a minor role in CBT. However, the body of research on psychodynamic and relational approaches which focus on interaction, transference and relational aspects indicates that these, too, can be effective via remote psychotherapy (Gordon et al., 2015; Dennis et al., 2020).

Markowitz et al. (2021) point out that, prior to the onset of the COVID-19 pandemic, research on remote psychotherapy was frequently conducted with selected populations (e.g., HIV-positive patients, war-veterans or women with postpartum depression in rural areas), often as an adjunct to in-person psychotherapy. The situation has fundamentally changed now. Wind et al. (2020) highlight that the present pandemic amounts to an unforeseen event which changes the ways we think, practice and research online mental-health care. A study by Boldrini et al. (2020) with psychotherapists of different orientations in Italy at the first peak of the pandemic in early 2020 unexpectedly shows CBT practitioners experiencing significantly more therapy interruptions than their psychodynamic colleagues when implementing remote psychotherapy. This comes as a surprise to the authors, since CBT practitioners had been deemed more up to task with remote psychotherapy and its implementation. A recent study by Humer et al. (2020) examined experiences of psychotherapists across four different psychotherapeutic orientations accredited in Austria: psychodynamic, humanistic-existential, systemic and behavioral. Interestingly, it appears that psychodynamic and humanistic psychotherapists had better experiences with remote psychotherapy than their behavioral or systemic colleagues.

In light of the above, remote psychotherapy is a credible and trustworthy alternative to be considered and adopted by psychotherapists regardless of their orientation. It provides mental-health care in times of crises such as the COVID-19 pandemic and allows for treatment and supervision when in-person contact is not possible due to large geographical distances (Markowitz et al., 2021). Furthermore, it increases accessibility for hard-to-reach patients who may not attend in-person sessions due to certain pathologies such as social anxiety or simply a tight schedule (Simpson et al., 2021). Reduced financial and time cost is another key point frequently mentioned in favor of remote psychotherapy (Poletti et al., 2020).

### Therapeutic Relationship

Therapeutic alliance and relationship are crucial factors for the effectiveness of the therapeutic process (Wampold and Imel, 2015). The carefully handled therapeutic relationship is an indispensable prerequisite for specific interventions and techniques such as transference interpretation, exposure or desensitization (Norcross and Lambert, 2019). In a systematic review, Sucala et al. (2012) point out that remote psychotherapy seems to be equivalent to in-person treatment in terms of therapeutic alliance as part of the psychotherapeutic relationship. Simpson et al. (2021) reviewed a number of studies showing that the quality of crucial factors of the psychotherapeutic relationship such as empathy and working alliance was not significantly different in remote psychotherapy compared to in-person treatment.

However, psychotherapists who suddenly had to deliver remote psychotherapy without training during the COVID-19 pandemic have reported challenges and constraints in establishing and maintaining the therapeutic relationship: Feelings of isolation in sessions, technical problems, difficulties maintaining the therapeutic attitude, rapid fatigue as well as feelings of lack of self-confidence and effectiveness (Békés and van Doorn, 2020; McBeath et al., 2020; Höfner et al., 2021a; Messina and Löffler-Stastka, 2021). According to MacMullin et al. (2020), reduced sensory perception of the person, the situation and the patient’s whole body, could pose a risk to the therapeutic relationship in emotionally charged situations. Markowitz et al. (2021) indicate that some patients perceive video therapy as an invasion of their privacy, that remote psychotherapy as a whole lacks the safe-space setting outside of the patients’ own, sometimes-precarious living situations. Furthermore, distractions and disturbances caused by family members may occur and the lack of warm-up and cool-down phases when traveling to and from the clinician’s office may impair the therapeutic process.

Conversely, it has been shown that in remote psychotherapy some patients are able to be more open and feel safer, they may perceive the setting in front of the screen in their own familiar environment as more at eye level and less confrontational (Simpson et al., 2021). According to the authors, evidence suggests that for some patient groups, e.g., those with anxious-avoidant personality structure, for whom in-person contact is overwhelming, remote psychotherapy yields better results than in-person treatment. Even though psychotherapists experienced some professional self-doubt or anxiety and worry about technicalities and therapeutic relationship in the early phase of the pandemic in 2020, they reported a relatively good working alliance and strong real relationship with their patients in a remote setting (Aafjes-van Doorn et al., 2020). Despite reports of more directive and talkative behavior, a study by Mancinelli et al. (2021) shows an overall positive self-perception in psychotherapists.

## Materials and Methods

### Study Design

Three test periods were set with the first (t1: April, 6th–April, 30th 2020) during the first lockdown in spring 2020, the second (t2: May, 12th–June, 14th 2020) when restrictions were lifted and the third (t3: November, 20th–December, 19th 2020) when lockdown came into force again in fall 2020 due to the second wave of COVID-19 infections. Psychotherapists of the Austrian Association for Group Therapy and Group Dynamics (ÖAGG) were sent a link to an online survey via SoSciSurvey. This survey contained a combination of 55 open and closed questions addressing fears and concerns of participants and their experiences with the transition to remote psychotherapy. Items and questions were developed by the authors of the present study. In addition, standardized questionnaires to assess quality of life (WHOQOL-BREF; Angermeyer et al., 2000), resilience (CD-RISC-10; Sarubin et al., 2015), and affectivity (PANAS; Janke and Glöckner-Rist, 2014) were included. The survey was conducted in German language and subsequently translated for the present paper. The study was analyzed using a combination of quantitative and qualitative approaches. The addition of open questions enabled the research team to gather further information on psychotherapists’ individual experiences which might have been overlooked in a purely quantitative study. The questionnaire remained unchanged over the first two test periods. For the third test period, some questions were removed and those asking for “experiences over the last 3 weeks” were changed to ask for “experiences from November 2nd, 2020, onward” in order to specifically explore experiences of the November 2020 lockdown. In Austria, remote psychotherapy was not implemented in the health-care system until the pandemic emerged; thus, the study didn’t examine experiences with this modality before the transition.

The survey’s design allowed for the collection of a wide range of sociodemographic and other variables such as age (in 5-year categories), sex, marital status, main residence, highest level of education, psychotherapeutic experience and orientation as well as type and extent of employment before and during the COVID-19 pandemic. As the pandemic and accompanying restrictions presented an exceptional situation, data regarding psychotherapists’ personal wellbeing were gathered, including questions around their activities, emotions, thoughts and general health. In addition, the survey asked participants to assess thematic changes and experiences with specific work techniques in remote treatment, as well as gathering information on the number of hours worked, changes to patient numbers and sociodemographic variables regarding their patients. The items comprised of check boxes and scales of 1–5, several open questions for qualitative analysis were posed, allowing participants to type in their answers. The present paper focuses on psychotherapists’ experiences with the transition and changes to the therapeutic relationship, whilst other aspects of the study have been published separately by Höfner et al. (2021a; 2021b) and Mantl et al. (2021).

### Ethical and Legal Considerations

Participation in the survey was voluntary, confidential and anonymous, and could be discontinued at any time without disadvantage. Participants were informed of the purpose of the present research project. The authors could be contacted in case of difficulties completing the survey, however, none of the participants made use of this offer. The data collected was stored and analyzed electronically in accordance with the legal requirements. All researchers able to access the data were subject to the Data Protection Regulation (DSGVO) and its currently valid Austrian adaptation. Data was not passed on to third parties or countries outside the EU. Participants were made aware of the estimated time required to complete the questionnaire. In order to proceed with the survey, they had to confirm they were over the age of 18 and consent to the use of their data as outlined above. The participants provided their written informed consent to participate in this survey. In accordance with the local legislation and institutional requirements, no further ethical review or approval was required for the present study.

### Participant Demographics

Currently, 23 psychotherapy methods are accredited in Austria. They comprise of four overarching orientations: psychodynamic, humanistic, systemic and behavioral (BMSGPK 2020). Compared to the distribution across Austria, the humanistic orientation is overrepresented in the present study over all three test periods with a participation rate of over 69%. The survey was administered to psychotherapists of the Austrian Association for Group Therapy and Group Dynamics (ÖAGG) and behavioral therapists are not part of this professional association. Thus, behavioral therapists did not participate in the present study.

175 online questionnaires were completed in full for the first test period t1, 79.4% of participants identify as female, 20.6% as male. 54 participants (30.9%) were still in training under supervision at the time of the survey. 177 online questionnaires were completed in full for the second test period t2. 79.1% of participants identify as female, 20.9% as male. 57 participants (32.2%) were still in training under supervision at the time of the survey. 113 online questionnaires were completed in full for the third test period t3. 77.0% of participants identify as female, 23.0% as male. 20 participants (17.7%) were still in training under supervision at the time of the survey. 25 psychotherapists who participated across all three test periods were identified based on the correlation and repetition of certain criteria (gender, age group, federal state, education, marital status, psychotherapeutic orientation). Of these, 76.0% of participants identify as female, 24.0% as male, 6 participants (24.0%) were still in training under supervision at the time of the survey. For further details on the therapist characteristics (see Table 1).

**TABLE 1:** Selected sociodemographic variables of the psychotherapists.

		t1		t2		t3	
Variable		*N*	%	*N*	%	*N*	%
Sex	Female	139	79.4	140	79.1	87	77.0
	Male	36	20.6	37	20.9	26	23.0
	Diverse	0	0	0	0	0	0
Age	25–29 years	2	1.1	0	0	0	0
	30–34 years	8	4.6	5	2.8	4	3.5
	35–39 years	22	12.6	16	9.0	13	11.5
	40–44 years	29	16.6	25	14.1	11	9.7
	45–49 years	24	13.7	23	13.0	17	15.0
	50–54 years	33	18.9	25	14.1	19	16.8
	55–59 years	29	16.6	44	24.9	25	22.1
	60–64 years	12	6.9	21	11.9	15	13.3
	>64 years	16	9.1	18	10.2	9	8.0
Psychotherapeutic orientation	Psychodynamic	18	10.3	15	8.5	11	9.7
	Humanistic	122	69.7	132	74.6	82	72.6
	Systemic	32	18.3	25	14.1	17	15.0
	Missing entry	3	1.7	5	2.8	3	2.7
Year of approbation	Under supervision before approbation	54	30.9	57	32.2	20	17.7
	1–11 years	59	33.7	49	27.7	53	46.9
	12–23 years	31	17.7	33	18.6	15	13.3
	>23 years	31	17.7	38	21.5	25	22.1

*Sociodemographic variables; t1–t3, test periods; N, sample size; %, percentage of participants.*

### Quantitative Analysis—Experiences With the Transition

#### Statistics

The quantitative analyses were computed with SPSS 18.0. To measure experiences with the transition, psychotherapists were asked for their perceived levels of experience with remote psychotherapy on a scale of 1–5, with 1 representing minimal experience and 5 representing maximal experience. The same scale was used to ascertain the perceived level of experience in the use of individual types of media for remote psychotherapy. Medians were calculated based on the ordinal scale level. Since the requirements for the analysis of variance were not met, Friedman tests were used to verify if the central tendencies of the dependent samples t1, t2 and t3 differed. Based on significant differences, subsequent *post hoc* tests were applied using the asymptotic Wilcoxon test and Cohen’s (1992) *d* calculations as a measure of effect size. For all analyses, the significance level was set at *p* ≤ 0.05. A within-subject design was chosen. With regard to certain criteria (gender, age group, federal state, education, marital status, psychotherapeutic orientation) after completion of the surveys, 25 matching cases from t1, t2, and t3 could be manually identified in terms of a measurement repetition and were subsequently used in the statistical analyses.

#### Results

The vast majority of participants transitioned to remote psychotherapy at the onset of the COVID-19 pandemic, with 92% of respondents reporting the use of remote psychotherapy to treat patients at t1 and 75.1% at t2. When restrictions were lifted in May and June 2020, a large proportion of therapists continued its use, with a further increase to 85% at t3 in November and December 2020. Results show psychotherapists feeling “very experienced” in delivering remote psychotherapy at t1 (*M* = 3.75, *SD* = 1.03), at t2 (*M* = 3.90; *SD* = 1.02) and at t3 (*M* = 3.88, *SD* = 1.02) with a median of 4. A slight increase in level of experience appears from t1 to t2 and t3. There is no statistically significant difference when considering the measured values from the 25 matching cases identified [Friedman test: χ*^2^*(2) = 0.59, *p* = 0.747, *N* = 25].

Examining differences in psychotherapists’ levels of experience offering telephone-based psychotherapy at all three test periods, results show them feeling “extremely experienced” at t1 (*M* = 4.26, *SD* = 0.99), at t2 (*M* = 4.37, *SD* = 0.77) and at t3 (*M* = 4.28, *SD* = 0.94) with a median of 5 at all three test periods. There appears to be no statistically significant variation regarding the level of experience delivering telephone-based psychotherapy across the test periods [Friedman test: χ*^2^*(2) = 4.44, *p* = 0.109, *N* = 25].

The results regarding video therapy show psychotherapists feeling only “rather experienced” at t1 (*M* = 2.95, *SD* = 1.30) and at t2 (*M* = 3.28, *SD* = 1.18), with a median of 3. At t3 (*M* = 3.38, *SD* = 1.15) psychotherapists perceive themselves as “experienced” in video therapy with a median of 4. Over the period of t1 and t2 as well as between t1 and t3, a slight increase in the perceived level of experience was reported. No statistically significant difference was observed when considering the measured values from the 25 matching cases identified [Friedman test: χ*^2^*(2) = 1.49, *p* = 0.476, *N* = 25].

Upon examination of participants’ level of experience using laptop or desktop computers, tablets or iPad, results show that at t1 (*M* = 3.61, *SD* = 1.14), at t2 (*M* = 3.93, *SD* = 0.93) and at t3 (*M* = 3.81, *SD* = 1.00) the respondents feel “experienced” with a median of 4 at all three test periods. There was no statistically significant difference in the use of laptop or desktop computers, tablets or iPad across the test periods [Friedman test: χ^2^(2) = 2.35, *p* = 0.309, *N* = 25].

**FIGURE 1:**
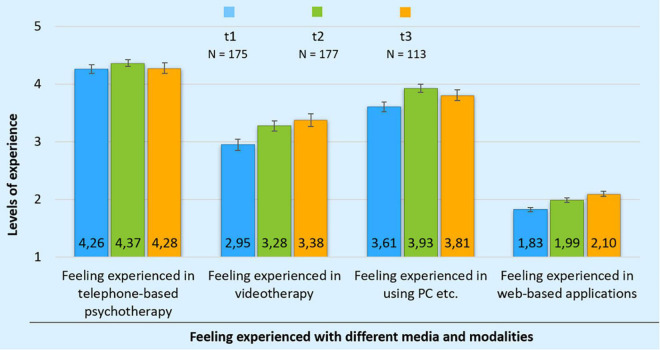
Changes to the perceived level of experience with different media and modalities during the transition over all three test periods. Value “1” represents complete inexperience and value “5” an extremely high level of experience. N: sample size; t1–t3 test periods.

Regarding the level of experience using web-based applications (Skype, Zoom, Facetime, WhatsApp, Signal, TheraPsy Connect, Instahelp, Telegram, Threema, fair-meeting, Jitsi Meet), the descriptive statistics show the participants feeling inexperienced at t1 (*M* = 1.83, *SD* = 0.47), slightly more experienced at t2 (*M* = 1.99, *SD* = 0.52) and at t3 (*M* = 2.10, *SD* = 0.51). With a median of 2, participants feel “inexperienced” with web-based applications across all test periods. The increasing trend shows a statistically significant difference when considering the measured values from the 25 matching cases identified [Friedman test: χ^2^(2) = 6.71, *p* = 0.035, *N* = 25]. The level of experience using these specific applications is significantly higher at t3 than at t1. Subsequent *post hoc* tests show that the level of experience using these specific applications is significantly higher at t3 than at t1 (asymptotic Wilcoxon test: *z* = –2.70, *p* = 0.007, *N* = 25). The statistical effect size is Cohen’s (1992) *d* = 1.28, corresponding to a large effect. There is no significant difference in psychotherapists’ experience with these specific apps between t1 and t2 (asymptotic Wilcoxon test: *z* = –1.95, *p* = 0.051, *N* = 25) or t2 and t3 (asymptotic Wilcoxon test: *z* = –1.54, *p* = 0.125, *N* = 25). Table 2 presents these results. Figure 1 illustrates changes to the perceived level of experience with different media and modalities.

**TABLE 2:** Changes to the perceived level of experience with remote psychotherapy, different media and modalities during the transition over all test periods.

	t1			t2			t3			Changes t1–t3			
Item	*N*	*M*	*(SD)*	*N*	*M*	*(SD)*	*N*	*M*	*(SD)*	χ^2^	*p*	*| d |*	*N*
Perceived level of experience delivering remote psychotherapy	175	3.75	(1.03)	177	3.90	(1.02)	113	3.88	(1.02)	0.59	0.747	–	25
Level of experience delivering telephone-based psychotherapy	175	4.26	(0.99)	177	4.37	(0.77)	113	4.28	(0.94)	4.44	0.109	–	25
Level of experience delivering video therapy	175	2.95	(1.30)	177	3.28	(1.18)	113	3.38	(1.15)	1.49	0.476	–	25
Level of experience using PC etc.	175	3.61	(1.14)	177	3.93	(0.93)	113	3.81	(1.00)	2.35	0.309	–	25
Level of experience using web-based applications	175	1.83	(0.47)	177	1.99	(0.52)	113	2.10	(0.51)	6.71	0.035*	1.281^1^	25

*The Psychotherapists’ perceived levels of experience with remote psychotherapy were measured on a scale of 1–5, with 1 representing minimal experience and 5 representing maximal experience. Therefore, the means only could assume values between 1 and 5. N, sample size. M, mean. SD, standard deviation. t1–t3, test periods.*

p, Significance level. *Significant mean difference p < 0.05. | d |, Cohen’s d.

*^1^Large effect between t1 and t3.*

### Qualitative Analysis—Changes in the Therapeutic Relationship

#### Qualitative Content Analysis

Perceived changes in the therapeutic relationship were explored via the open question “Which changes in the therapeutic relationship do you perceive with the use of remote psychotherapy?” Some participants chose to respond in complete sentences, while others used keywords, phrases or lists.

The text content was analyzed by means of Qualitative Content Analysis (Mayring, 2015). The distinguishing feature of this method is its research-question-oriented procedure with a category-based approach, which additionally allows for quantitative analysis when required. Categories refer to certain aspects of the text analyzed, based on common denominators within the content of these aspects (Mayring, 2019). Several techniques for evaluation may be applied within the framework, the present study used a combination of structuring and inductive category formation. The software tool ATLAS.ti. 8.0 was used to process participants’ responses, supporting the development of categories using systematic coding. The initial step comprised the deduction of central factors in the psychotherapeutic relationship based on the findings outlined in the “State Of The Art” section of the present paper in order to structure the content. Considering these points of reference, changes of the quality of the therapeutic relationship and perceived constraints handling the therapeutic relationship appeared to be the most important issues to the authors of the present study.

Based on this initial structure, inductive category formation was used to analyze the data. Subcategories were developed to expand the category system accordingly. In order to define inductive subcategories, the text material was analyzed line by line to see which concepts stood out and were repeated in the text. From the resulting lists of concepts, further categories were developed inductively, with statements of similar content subsumed in the respective categories. Each category was labeled with a term or short phrase highlighting the content. Any responses not suited for assignment to an existing category led to further expansion of the categories used. Answers not fitting any subcategories were grouped into the category “other”. Ultimately, three main categories were formed: (1) Changes in the perceived quality of the psychotherapeutic relationship, comprising five subcategories; (2) Perceived constraints handling the therapeutic relationship, comprising four subcategories; (3) Summarized answers that did not indicate any changes to the psychotherapeutic relationship (see Table 3).

**TABLE 3:** Main categories, selected subcategories and numbers for each test period t1–t3.

Main category	Subcategory	t1	t2	t3
Quality of the therapeutic relationship changes		90	92	63
	Psychotherapeutic relationship intensifies	24	26	9
	Patients are able to open up more	4	9	7
	Psychotherapeutic relationship feels more distant	16	14	17
	Psychotherapeutic relationship feels more superficial	12	9	9
	Other	34	34	21
Perceived constraints handling therapeutic relationship		80	42	64
	Full-sensory perception of the person and situation	22	9	11
	Empathizing with the patient	5	2	9
	Establishing and maintaining contact	4	4	1
	Technical problems	9	2	6
	Rapid fatigue and exhaustion	6	2	4
	Other	34	23	29
No changes of the therapeutic relationship		29	23	30

#### Results

Responses across the three test periods reported changes in the quality of the therapeutic relationship (t1: 90, t2: 92, t3: 63). A considerable number of responses indicated an increased intensity in the psychotherapeutic relationship at the onset of the COVID-19 pandemic and the transition to remote psychotherapy. However, a substantial decrease in intensity toward time period three (t1: 24, t2: 26, t3: 9) was noted. Some answers suggest that patients were able to open up more easily when speaking to their psychotherapist (t1: 4, t2: 9, t3: 7).

“For some people it even seems to be a relief to talk on the phone, different topics which are very shameful arose and thus became addressable”, one psychotherapist wrote.

Psychotherapists continuously experienced a feeling of distance (t1: 16, t2: 14, t3: 17) and the psychotherapeutic relationship via remote psychotherapy was reported to feel more superficial (t1: 12, t2: 9, t3: 9).

“By using online tools, I think there is a greater emotional distance.”, the relationship is “much harder to deepen, remains superficial.”

A sense of togetherness based on the shared experience of the pandemic seemed to be important with the onset of COVID-19 but vanished with test period three (t1: 8, t2: 4, t3: 0). Nevertheless, the use of remote psychotherapy does preserve the psychotherapeutic relationship beyond mere keeping in touch, although a decrease in responses to that effect was noted (t1: 6, t2: 4, t3: 2). Psychotherapists also reported changes to their style of working across all three test periods:

“As a therapist, I often experience myself as ‘overly active’ to the point of just ‘giving advice’.”

During test period two, some responses indicated that therapists were very much looking forward to a return to in-person treatment (t1: 0, t2: 7, t3: 0). A few participants noticed an increase in patients’ concern for their therapist’s wellbeing in the early stages of the pandemic. An increased inhibition or restraint on the patient’s side was reported in some cases. A small number of responses indicated a reduced commitment in their patients, which was attributed to the use of remote psychotherapy. Occasionally, psychotherapists noticed a distinct change in the quality of the psychotherapeutic relationship at test periods one and two, reporting a degree of regression in their patients. Across the three test periods, psychotherapists frequently reported differences in psychotherapeutic relationship but no clear definition of the term “different” was given in many of the responses.

Across all three test periods, respondents experienced constraints regarding the psychotherapeutic relationship (t1: 80, t2: 42, t3: 64). These were primarily attributed to a decreased sensory perception (t1: 22, t2: 9 t3: 11) and difficulties to properly empathize with their patients during remote treatment (t1: 5, t2: 2, t3: 9). Psychotherapists found it harder to get to know their patients and maintain contact when using remote psychotherapy. One participant noted, “Building rapport is clearly more difficult.” Test period three showed a decrease in the difficulty establishing contact (t1: 4, t2: 4, t3: 1). Participants attributed the perceived constraints to the lack of a physical encounter (t1: 2, t2: 1, t3: 5). “These tools never replace real contact”, one psychotherapist wrote. Difficulties regarding initial interviews and keeping up the flow of the conversation were reported. Technical issues were perceived as potentially harmful to the quality of the therapeutic relationship and led to stress in treatment (t1: 9, t2: 2, t3: 6):

“Because of poor technical connection, [I] stress when establishing the connection”, one psychotherapist reported.

Some participants experienced difficulties establishing the psychotherapeutic process, reporting an increased effort on their side. Few answers indicated an increased risk of therapy discontinuation at test period three (t1: 0, t2: 0, t3: 2). Occasionally, constraints were experienced in psychotherapy with children. Some responses pointed to the risk of not being able to pay sufficient attention due to the use of remote technologies. A few respondents mentioned blurred roles within remote psychotherapy and found it harder to cope with silence when on the phone compared to in-person treatment. Respondents continuously noted increased difficulties regarding their own mental and physical functioning (t1: 6, t2: 2, t3: 4). Some symptoms described were fatigue, eye pain or headaches. One participant commented on remote psychotherapy in comparison to in-person treatment: “I find it exhausting.”

A considerable number of respondents reported no changes in the psychotherapeutic relationship due to the use of remote psychotherapy (t1: 29, t2: 23, t3: 26).

## Discussion

Remote psychotherapy might pose a challenge to the psychotherapeutic relationship and setting, particularly when it suddenly is the only option for treatment (Aafjes-van Doorn et al., 2020; Békés and van Doorn, 2020; Crowe et al., 2021; Höfner et al., 2021a; Messina and Löffler-Stastka, 2021). The longitudinal effects of a forced provision of remote psychotherapy were the primary interest of the present study. The vast majority of participants started to deliver remote psychotherapy with the onset of the COVID-19 pandemic. When restrictions were lifted at test period t2, most respondents continued to work remotely. At test period t3 in November and December 2020, with lockdown in effect again, the use of remote psychotherapy slightly increased, even though it was legally possible to offer in-person treatment in Austria at that time. Whether this was due to health concerns or remote psychotherapy being perceived as an effective means of treatment is beyond the scope of the present study. While the perceived proficiency in telephone-based psychotherapy remains relatively stable across all test periods, psychotherapists feel slightly more experienced with video therapy over the test period observed. However, psychotherapists remain less experienced using video therapy compared to telephone-based psychotherapy, which comes as no surprise. Telephone-based communication is often used in psychotherapeutic crisis intervention, therefore many therapists were familiar with it prior to the pandemic. Despite a significant improvement from t1 to t3, participants still feel rather inexperienced using web-based applications across all test periods. This might be cause for concern since a large proportion of remote psychotherapy is now delivered via web-based applications and videoconferencing tools in particular (Markowitz et al., 2021).

In line with previous findings (Sucala et al., 2012; Simpson et al., 2021), no general tendency toward a deterioration of the quality of the therapeutic relationship due to remote psychotherapy can be observed in the current study. This is relevant, as it disproves some prejudices critically discussed by Wind et al. (2020), particularly the notion that the therapeutic alliance can only be established in in-person treatment. Simpson et al. (2021) point out that some patients feel safer and may even talk more openly with remote psychotherapy. The present study shows this to be the case, too. Remarkably, the quality of the therapeutic relationship seems to improve during the first two test periods. Positive and negative changes in the psychotherapeutic relationship were reported in roughly equal amounts for test period t1 in the qualitative part of the survey. In the second test period t2, positive changes outweighed the negative. As some answers suggest, the mutual experience of clinician and patient going through the pandemic may have intensified the psychotherapeutic relationship; the shared outlook of getting through this together during the first lockdown in Spring 2020 might have contributed to this phenomenon. Nevertheless, these longitudinal findings are encouraging compared to cross-sectional surveys conducted at the first peak of the pandemic (Aafjes-van Doorn et al., 2020; Békés and van Doorn, 2020; McBeath et al., 2020). Surprisingly, many answers at t1 but only a few at t2 indicate that psychotherapists suffer from constraints regarding full-sensory perception of their patients during remote psychotherapy sessions. Psychotherapists seem to adapt and enhance their skills in remote psychotherapy over time, as Mancinelli et al. (2021) have similarly observed in Italian psychotherapists during the pandemic. With curfews imposed again at test period t3 in Fall 2020, the previously positive attitude changed. More constraints in handling the psychotherapeutic relationship were reported and the intensity of the therapeutic relationship seemed to slightly decrease, being perceived as becoming more superficial. This could indicate a time limit regarding the possibility of maintaining a therapeutic relationship via remote therapy, especially with psychotherapists very much untrained in this modality. In addition, from an affective neuroscience perspective, perceived physical distance has an impact on empathic reactions (Schiano Lomoriello et al., 2018), making it difficult to maintain the relationship over time, which could be the reason why participants in the present study found it hard to be empathic with the patient during the last time period t3. As pointed out by Cao et al. (2020) and Boldrini et al. (2021), psychosocial sequelae of the COVID-19 pandemic had a considerable impact on society and thus on clinicians and patients, presumably making it harder to keep up with the therapeutic relationship in Fall 2020. It is up to future research to determine if and how this could be improved by more specific training and supervision in remote psychotherapy, especially under non-pandemic conditions.

## Limitations

A number of limitations in this study need to be addressed. The selection of psychotherapists could be a potential source of bias, as no representative sample was collected. Research conducted using online surveys may always be biased because psychotherapists who are open to electronic data processing and the use of online tools tend to participate (Markowitz et al., 2021). Accordingly, they may report a more positive experience and feedback on remote psychotherapy compared to a representative sample of psychotherapists. The link to the questionnaire was only administered to psychotherapists of the Austrian Association for Group Therapy and Group Dynamics (ÖAGG) via e-mail. The ÖAGG comprises of psychodynamic, humanistic-existential and systemic psychotherapists. Compared to the Austrian distribution as a whole, the humanistic-existential orientation was overrepresented and no behaviorally oriented psychotherapists took part. Another limitation regarding the analysis of the results was that no data are available on the situation prior to the involuntary transition to remote psychotherapy with the present sample. Furthermore, compared to t1 (*N* = 175) and t2 (*N* = 177), fewer responses were received with t3 (*N* = 113). This might be cause for bias, meaning the number of responses in the qualitative part in particular must be interpreted in light of this for t3. In a further limitation, the participants’ ages were disregarded when evaluating their experience of web-based applications. No additional demographic data such as age or sex were controlled for the results in the analyses.

## Conclusion

To conclude, psychotherapists of different orientations seem well able to meet the challenges of delivering remote psychotherapy when it is the only option. The current results confirm and enhance previous findings: Remote psychotherapy can be a credible and trustworthy alternative to in-person treatment to be adopted and implemented on principle by a majority of psychotherapists regardless of their orientation. However, difficulties described in literature, such as establishing and maintaining the therapeutic relationship (Cataldo et al., 2021) have been observed in the present study. Constraints regarding full-sensory perception and technical issues might play a considerable role in this, as well as problems with exhaustion or rapid fatigue, remaining attentive in front of a screen and missing physical encounter, as frequently reported in previous research (Békés and van Doorn, 2020; McBeath et al., 2020; Markowitz et al., 2021). Fortunately, psychotherapists seem to adjust and grow more comfortable over time when delivering remote treatment. This indicates that better training and education regarding remote therapy would enable psychotherapists to handle these challenges and use electronic media more confidently (Connolly et al., 2020; Grondin et al., 2020). Ultimately, this would also benefit patients, as it has been frequently shown that self-confidence and positive self-perception on the psychotherapist’s side correlates with positive treatment outcome (Wampold and Imel, 2015). Psychotherapists need to continue to adapt but also require specific support measures from health care stakeholders and training institutions so that high quality treatment can be achieved.

## Data Availability Statement

The datasets presented in this article are not readily available because data are subject to the Data Protection Regulation (DSGVO). Requests to access the datasets should be directed to corresponding author.

## Ethics Statement

Ethical review and approval was not required for the study on human participants in accordance with the local legislation and institutional requirements. The patients/participants provided their written informed consent to participate in this study.

## Author Contributions

CH, GM, and MH: conceptualization. GM, JS, and CH: methodology, formal analysis, investigation, and data curation. RS: writing—original draft preparation. RS, GM, and KP: writing—review and editing. All authors have read and agreed to the published version of the manuscript.

## Conflict of Interest

The authors declare that the research was conducted in the absence of any commercial or financial relationships that could be construed as a potential conflict of interest.

## Publisher’s Note

All claims expressed in this article are solely those of the authors and do not necessarily represent those of their affiliated organizations, or those of the publisher, the editors and the reviewers. Any product that may be evaluated in this article, or claim that may be made by its manufacturer, is not guaranteed or endorsed by the publisher.

## References

[B1] Aafjes-van Doorn K. Békés V. Prout T. A. (2020). Grappling with our therapeutic relationship and professional self-doubt during COVID-19: will we use video therapy again? *Couns. Psychol. Q.* 11 1–12. 10.1080/09515070.2020.1773404

[B2] Angermeyer M. C. Kilian R. Matschinger H. (2000). *WHOQOL-100 und WHOQOL-BREF - Handbuch Für Die Deutschsprachigen Versionen der WHO Instrumente zur Erfassung von Lebensqualität.* Göttingen: Hogrefe.

[B3] BMSGPK (2020). *PatientInnen/Patienten-Information Über die in Österreich Anerkannten Psychotherapeutischen Verfahren.* Available online at: https://www.sozialministerium.at/dam/jcr:067ed3c8-aaea-4c84-84c2-a3afb9cef836/Patienteninformation_(BMGSPK),_Stand_29.04.2020.pdfAbg (accessed December 20, 2020).

[B4] Barak A. Grohol J. M. (2011). Current and future trends in internet-supported mental health interventions. *J. Technol. Hum. Serv.* 29 155–196. 10.1080/15228835.2011.616939

[B5] Békés V. van Doorn K. A. (2020). Psychotherapists’ attitudes toward online therapy during the COVID-19 pandemic. *J. Psychother. Integr.* 30 238–247. 10.1037/int0000214

[B6] Boldrini T. Girardi P. Clerici M. Conca A. Creati C. Di Cicilia G. (2021). Consequences of the COVID-19 pandemic on admissions to general hospital psychiatric wards in Italy: reduced psychiatric hospitalizations and increased suicidality. *Prog. Neuropsychopharmacol. Biol. Psychiatry* 110:110304. 10.1016/j.pnpbp.2021.110304 33737215 PMC8569419

[B7] Boldrini T. Schiano Lomoriello A. Del Corno F. Lingiardi V. Salcuni S. (2020). Psychotherapy during COVID-19: how the clinical practice of Italian psychotherapists changed during the pandemic. *Front. Psychol.* 11:591170. 10.3389/fpsyg.2020.591170 33192932 PMC7641613

[B8] Cao W. Fang Z. Hou G. Han M. Xu X. Dong J. (2020). The psychological impact of the COVID-19 epidemic on college students in China. *Psychiatry Res.* 287:112934. 10.1016/j.psychres.2020.112934 32229390 PMC7102633

[B9] Carlbring P. Andersson G. Cuijpers P. Riper H. Hedman-Lagerlöf E. (2018). Internet-based vs. face-to-face cognitive behavior therapy for psychiatric and somatic disorders: an updated systematic review and meta-analysis. *Cogn. Behav. Ther.* 47 1–18. 10.1080/16506073.2017.1401115 29215315

[B10] Cataldo F. Chang S. Mendoza A. Buchanan G. (2021). A perspective on client-psychologist relationships in videoconferencing psychotherapy: literature review. *JMIR Mental Health*. 8:e19004. 10.2196/19004 33605891 PMC7935652

[B11] Cohen J. (1992). Statistical power analysis. *Curr. Dir. Psychol. Sci.* 1 98–101. 10.1111/1467-8721.ep10768783

[B12] Connolly S. L. Miller C. J. Lindsay J. A. Bauer M. S. (2020). A systematic review of providers’ attitudes toward telemental health via videoconferencing. *Clin. Psychol. Sci. Pract*. 27:e12311. 10.1111/cpsp.12311 PMC9367168 35966216

[B13] Crowe M. Inder M. Farmar R. Carlyle D. (2021). Delivering psychotherapy by video conference in the time of COVID-19: some considerations. *J. Psychiatr. Ment. Health Nurs.* 28 751–752. 10.1111/jpm.12659 32413164

[B14] Dennis C. Grigoriadis S. Zupancic J. Kiss A. Ravitz P. (2020). Telephone-based nurse-delivered interpersonal psychotherapy for postpartum depression: nationwide randomised controlled trial. *Br. J. Psychiatry.* 216 189–196. 10.1192/bjp.2019.275 32029010

[B15] Gordon R. M. Wang X. Tune J. (2015). Comparing psychodynamic teaching, supervision, and psychotherapy over videoconferencing technology with Chinese students. *Psychodyn. Psychiatry*. 43 585–599. 10.1521/pdps.2015.43.4.585 26583442

[B16] Grondin F. Lomanowska A. M. Békés V. Jackson P. L. (2020). A methodology to improve eye contact in telepsychotherapy via videoconferencing with considerations for psychological distance. *Couns. Psychol. Q.* 10.1080/09515070.2020.1781596. [Epub ahead of print].

[B17] Höfner C. Hochgerner M. Mantl G. Stefan R. Stammer J. (2021a). Telepsychotherapie als chance und herausforderung: eine longitudinale mixed-methods studie. *Psychother. Forum* 25 37–43. 10.1007/s00729-021-00169-2

[B18] Höfner C. Mantl G. Korunka C. Hochgerner M. (2021b). Psychotherapie in zeiten der Covid-19-pandemie: veränderung der arbeitsbedingungen in der versorgungspraxis. *Feedback* 1-2 23–38.

[B19] Humer E. Stippl P. Pieh C. Schimböck W. Probst T. (2020). Psychotherapy via the internet: what programs do psychotherapists use, how well-informed do they feel, and what are their wishes for continuous education? *Int. J. Environ. Res. Public. Health*. 17 8182. 10.3390/ijerph17218182 33167478 PMC7663907

[B20] Janke S. Glöckner-Rist A. (2014). *Deutsche Version der Positive andNegative Affect Schedule (PANAS). Zusammenstellung Sozialwissenschaftlicher Items und Skalen (ZIS)*. Available online at: https://zis.gesis.org/skala/Janke-Gl ckner-Rist-Deutsche-Version-der-Positive-and-Negative-Affect-Schedule-(PANAS)

[B21] Korecka N. Rabenstein R. Pieh C. Stippl P. Barke A. Doering B. (2020). Psychotherapy by telephone or internet in Austria and Germany which CBT psychotherapists rate it more comparable to face-to-face psychotherapy in personal contact and have more positive actual experiences compared to previous expectations? *Int. J. Environ. Res. Public. Health*. 17 7756. 10.3390/ijerph17217756 33114136 PMC7660328

[B22] MacMullin K. Jerry P. Cook K. (2020). Psychotherapist experiences with telepsychotherapy: pre COVID-19 lessons for a post COVID-19 world. *J. Psychother. Integr.* 30 248–264. 10.1037/int0000213

[B23] Mancinelli E. Gritti E. S. Schiano Lomoriello A. Salcuni S. Lingiardi V. Boldrini T. (2021). How does it feel to be online? Psychotherapists‘ self-perceptions in telepsychotherapy sessions during the COVID-19 pandemic in Italy. *Front. Psychol*. 12:726864. 10.3389/fpsyg.2021.726864 34539529 PMC8446272

[B24] Mantl G. Höfner C. Stammer J. Hochgerner M. (2021). Psychotherapie in der krise. eine längsschnittstudie zur lebens- und arbeitssituation von psychotherapeut*innen. *Feedback* 1-2 38–55.

[B25] Markowitz J. C. Milrod B. Heckman T. G. Bergman M. Amsalem D. Zalman H. (2021). Psychotherapy at a distance. *Am. J. Psychiatry.* 178 240–246. 10.1176/appi.ajp.2020.20050557 32972202

[B26] Mayring P. (2015). *Qualitative Inhaltsanalyse. Grundlagen und Techniken*, 12th Edn. Weinheim: Beltz.

[B27] Mayring P. (2019). Qualitative content analysis: demarcation, varieties, developments. *Forum Qual. Soc. Res.* 20 1–14.. 10.17169/fqs-20.3.3343

[B28] McBeath A. G. du Plock S. Bager-Charleson S. (2020). The challenges and experiences of psychotherapists working remotely during the coronavirus pandemic. *Couns. Psychother. Res.* 20 394–405. 10.1002/capr.12326 32837329 PMC7361364

[B29] Messina I. Löffler-Stastka H. (2021). Psychotherapists’ perception of their clinical skills and in-session feelings in live therapy versus online therapy during the COVID-19 pandemic: a pilot study. *Res. Psychother. Psychopathol. Process Outcome* 24 53–59. 10.4081/ripppo.2021.514 33937115 PMC8082529

[B30] Mohr D. C. Ho J. Duffecy J. Reifler D. Sokol L. Burns M. N. (2012). Effect of telephone-administered vs face-to-face cognitive behavioral therapy on adherence to therapy and depression outcomes among primary care patients: a randomized trial. *JAMA* 307 2278–2285. 10.1001/jama.2012.5588 22706833 PMC3697075

[B31] Mohr D. C. Vella L. Hart S. Heckman T. Simon G. (2008). The effect of telephone-administered psychotherapy on symptoms of depression and attrition: a meta-analysis. *Clin. Psychol.* 15 243–253. 10.1111/j.1468-2850.2008.00134 PMC3045729 21369344

[B32] Norcross J. C. Lambert M. J. (eds) (2019). *Psychotherapy Relationships That Work. Volume 1: Evidence- Based Therapist Contributions*, 3rd Edn. Oxford: Oxford University Press.

[B33] ÖBVP (2020). *Coronavirus – Informationen für PsychotherapeutInnen.* Avilable online at: https://www.psychotherapie.at/psychotherapeutinnen/coronavirus-informationen-psychotherapeutinnen#pthinternet (Accessed December12, 2020)

[B34] Ogden P. Goldstein B. (2020). “Sensorymotor psychotherapy from a distance: engaging body, creating presence, and building relationship in videoconferencing,” in *Theory and Practice of Online Therapy: Internet-delivered Interventions for Individuals, Families, Groups, and Organizations*, eds Weinberg H. Rolnick A. (New York, NY: Routledge), 47–63.

[B35] Poletti B. Tagini S. Brugnera A. Parolin L. Ferrucci R. Compare A. (2020). Telepsychotherapy: a leaflet for psychotherapists in the age of COVID-19. a review of the evidence. *Couns. Psychol. Q.* 10.1080/09515070.2020.1769557 [Epub ahead of print].

[B36] Probst T. Haid B. Schimböck W. Reisinger A. Gasser M. Eichberger-Heckmann H. (2021). Therapeutic interventions in in-person and remote psychotherapy: survey with psychotherapists and patients experiencing in-person and remote psychotherapy during COVID-19. *Clin. Psychol. Psychother.* 28 988–1000. 10.1002/cpp.2553 33448499 PMC8013388

[B37] Probst T. Humer E. Stippl P. Pieh C. (2020). Being a psychotherapist in times of the novel coronavirus disease: stress-level, job anxiety, and fear of coronavirus disease infection in more than 1,500 psychotherapists in Austria. *Front. Psychol.* 11:559100. 10.3389/fpsyg.2020.559100 33132965 PMC7550677

[B38] Sarubin N. Gutt D. Giegling I. Bühner M. Hilbert S. Krähenmann O. (2015). Erste analyse der psychometrischen eigenschaften und struktur der deutschsprachigen 10- und 25-item version der Connor-Davidson Resilience Scale (CD-RISC). *Zeitschrift für Gesundheitspsychologie* 23 112–122. 10.1026/0943-8149/a000142

[B39] Schiano Lomoriello A. Meconi F. Rinaldi I. Sessa P. (2018). Out of sight out of mind: perceived physical distance between the observer and someone in pain shapes observer’s neural empathic reactions. *Front. Psychol.* 9:1824. 10.3389/fpsyg.2018.01824 30364280 PMC6193079

[B40] Simpson S. Richardson L. Pietrabissa G. Castelnuovo G. Reid C. (2021). Videotherapy and therapeutic alliance in the age of COVID-19. *Clin. Psychol. Psychother*. 28 409–421. 10.1002/cpp.2521 33037682 PMC7675483

[B41] Sucala M. Schnur J. B. Constantino M. J. Miller S. J. Brackman E. H. Montgomery G. H. (2012). The therapeutic relationship in e-therapy for mental health: a systematic review. *J. Med. Internet. Res.* 14:e110. 10.2196/jmir.2084 22858538 PMC3411180

[B42] Swartz H. A. (2020). The role of psychotherapy during the COVID-19 pandemic. *Am. J. Psychother.* 73 41–42. 32516084 10.1176/appi.psychotherapy.20200015

[B43] Wampold B. E. Imel Z. E. (2015). *The Great Psychotherapy Debate. The Evidence for What Makes Psychotherapy Work.* New York, NY: Routledge.

[B44] Webster P. (2020). Virtual health care in the era of COVID-19. *Lancet.* 395 1180–1181. 10.1016/S0140-6736(20)30818-7 32278374 PMC7146660

[B45] Weinberg H. (2020). Online group psychotherapy: challenges and possibilities during COVID-19-A practice review. *Group Dyn.* 24 201–211. 10.1037/gdn0000140

[B46] Wind T. R. Rijkeboer M. Andersson G. Riper H. (2020). The COVID-19 pandemic: the “black swan” for mental health care and a turning point for e-health. *Internet Interv.* 20:100317. 10.1016/j.invent.2020.100317 32289019 PMC7104190

